# An uncharacterized gene from the Actinobacillus genus encodes a glucosyltransferase with successive transfer activity and unique substrate specificity

**DOI:** 10.1016/j.jbc.2025.108567

**Published:** 2025-04-30

**Authors:** Takahiro Yamasaki, Daisuke Kohda

**Affiliations:** 1 Glyco-Biochemistry Laboratory, Institute for Glyco-core Research (iGCORE), Gifu University, Gifu, Japan; 2 Division of Structural Biology, Medical Institute of Bioregulation, Kyushu University, Fukuoka, Japan

**Keywords:** glycosylation, glycosyltransferase, gram-negative bacteria, posttranslational modification (PTM), protein structure, X-ray crystallography

## Abstract

Elucidating the functions of glycosyltransferases is a necessary step toward understanding their biological roles and producing drug leads, cosmetics, and foods that utilize glycans as functional molecules. We found a previously uncharacterized protein classified as a glycosyltransferase encoded in the *Actinobacillus minor* NM305 genome and named the gene product *A. minor* glucoside-glucosyltransferase (AmGGT). To clarify the biochemical properties of the AmGGT protein, we determined its substrate specificity and crystal structure. AmGGT exhibited processive glycosyltransferase activity when UDP-Glc was used as the donor substrate and, unexpectedly, showed different acceptor substrate specificity from that of the homologous Agt proteins of other *Actinobacillus* species. While the homologous proteins transfer glucose residues to the nonreducing end of oligosaccharide chains linked to peptides, AmGGT cannot use glycopeptides as acceptors and requires the nonreducing end of oligosaccharides. The crystal structure provided clues to identify a sequence motif consisting of two pairs of two amino acid residues that defines the acceptor specificity, oligosaccharide, or glycopeptide. Based on this discovery, the acceptor substrate of AmGGT was changed from an oligosaccharide to a glycopeptide by transplanting the sequence motif from the homologous proteins. Furthermore, the AmGGT protein could utilize eukaryotic high-mannose type N-glycans as acceptors, as a model for branched oligosaccharides. The sequential glycosyltransfer activity and controllable substrate specificity of AmGGT will make it a useful tool in glycosyltransferase engineering to synthesize functional glycans and glycoconjugates.

Glycosylation is the transfer of monosaccharide and oligosaccharide to proteins, as well as metabolites, lipids, and other oligosaccharides (1). The covalent modification with monosaccharide and oligosaccharide increases the chemical complexity of the modified molecules (2). The attached sugars change the surface features, particularly the hydrophilicity, and thereby affect the molecular recognition and bioactivity of the glycosylated molecules. Studying glycosyltransferases facilitates applied research, including the development of vaccines and antibiotics against pathogenic bacteria and fungi, the production of useful rare sugars, and the improvement of the *in vivo* dynamics of foods and medicines (3, 4). Glycosyltransferase genes often belong to functional operons in bacterial genomes, greatly simplifying their functional prediction and characterization. Despite the clear benefits, many genes and gene clusters remain uncharacterized. For functional annotation of enzymes, 40% sequence identity is reportedly a confident threshold (5). However, oligosaccharides are often conjugated to various aglycone molecules, including proteins, lipids, and other organic compounds. Importantly, the structural diversity of aglycones plays a key role in generating functional variation in nature. We hypothesized that many glycosyltransferases may have wide substrate specificities not only for the oligosaccharide moiety but also the aglycones despite their highly conserved amino acid sequences. Biochemical approaches are useful to elucidate the functions of these putative glycosyltransferases. In the absence of operon information, structure determination provides clues for characterizing new glycosyltransferase proteins by structural similarity.

*Actinobacillus pleuropneumoniae* (*App*) is the pathogen of swine pleuropneumonia, and its infection causes economic damage to husbandry industries (6). In *App* cells, sequential glucose transfer occurs on autotransporter adhesin proteins in the cytoplasm (7). As a result, adhesin proteins escape recognition by the host immune system, which promotes the adhesion of *App* cells to the host cell surface. The glucosylation—*that is*, the addition of glucose residues—of the adhesin proteins is a two-step process (Fig. 1*A*). First, the *Actinobacillus pleuropneumoniae* UDP-Glc:protein *N*-β-glucosyltransferase (ApNGT) catalyzes the glucose moiety transfer from a nucleotide-activated sugar, UDP-Glc, to the asparagine residue in the consensus sequence (Asn-X-Ser/Thr, X≠Pro) to generate monoglucosylated proteins (8). This bacterial *N*-glycosylation is reminiscent of the eukaryotic *N*-glycosylation of proteins, although the bacterial and eukaryotic systems are evolutionarily different (9). Next, the accessory glucosyltransferase (ApAgt, also designated as α6GlcT) catalyzes the glucose moiety transfer from UDP-Glc to the terminal glucose residue, to elongate preexisting mono- and oligo-glucose chains on the adhesin proteins *via* an α1,6-glycosidic linkage. Deletion of the gene encoding either NGT or Agt reduced the rate of adhesion to the host cells (10). The ApAgt protein is a UDP-glucosyltransferase classified into the GT4 family in the carbohydrate active enzyme database (7). Unfortunately, detailed studies are limited to the NGT and Agt proteins derived from *App*, and the identification and functional characterization of homologous proteins from closely related species remain elusive.

**Figure 1:**
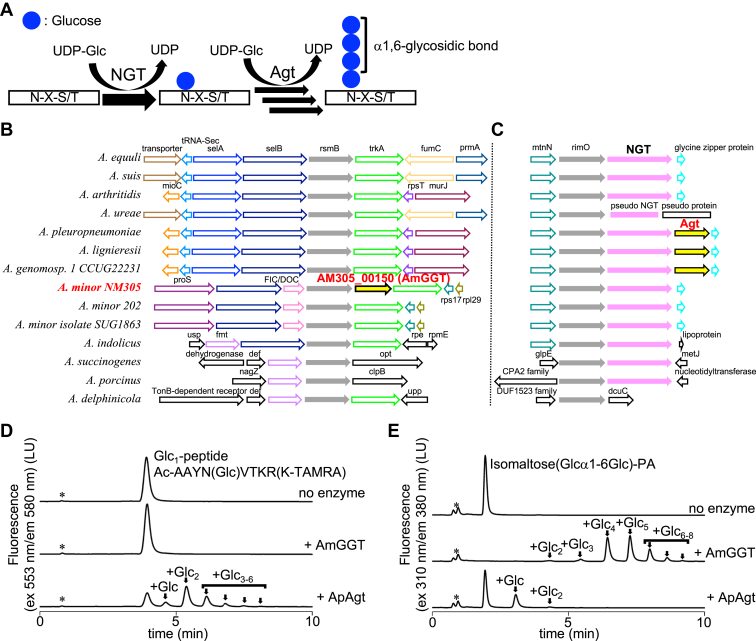
**Identification of a new UDP-glucosyltransferase in the *Actinobacillus minor* NM305 genome.***A*, schematic of the cytoplasmic *N*-linked glycosylation of proteins in *Actinobacillus pleuropneumoniae* cells. The ApNGT protein catalyzes the glucosyl transfer reaction to the asparagine residue in the consensus sequence, Asn-X-Ser/Thr, X≠Pro. The ApAgt protein then repeatedly adds a glucose residue to the asparagine-linked glucose chain. *B* and *C*, genetic compositions of 14 species in the *Actinobacillus* genus. The flanking regions of the rsmB and rimO genes are shown in (*B* and *C*), respectively. The genes are annotated by reference to the NCBI database. *D* and *E*, the AmGGT protein catalyzes the elongation of glucose residues with a different substrate specificity from that of the ApAgt protein. *Asterisks* indicate the positions of nonspecific peaks. *Arrows* indicate the enzymatic reaction products. In (*D*), the enzymatic activities of the recombinant AmGGT and ApAgt proteins were assayed using the glucose adduct of a fluorescent TAMRA-labeled peptide (Glc_1_-peptide) as the acceptor substrate. Reaction products were separated by normal-phase chromatography, and the fluorescence of the TAMRA fluorophore attached to the Glc_1_-peptide was monitored. In (*E*), the enzymatic activities of proteins were measured using isomaltose-PA (Glcα1-6Glc-PA) as the acceptor substrate. Reaction products were separated by normal-phase chromatography and the fluorescence of aminopyridine (AP) was monitored. AmGGT, *Actinobacillus minor* glucoside-glucosyltransferase; ApNGT, *Actinobacillus pleuropneumoniae* UDP-Glc:protein *N*-β-glucosyltransferase; PA, pyridyl amination.

Here, we focused on the AM305_00150 gene from the *Actinobacillus minor* NM305 strain. *A. minor* commonly inhabits the respiratory tracts of swine (11, 12). Our biochemical and structural analyses revealed that the AM305_00150 protein is a unique UDP-glucosyltransferase with different properties from the Agt proteins. Based on this finding, we named the AM305_00150 protein AmGGT (*A. minor* glucoside-glucosyltransferase).

## Results

### Mining of a putative glycosyltransferase gene in Actinobacillus genomes

The 16S rRNA sequences of 18 *Actinobacillus* strains were retrieved from the NCBI genome database and used to create a phylogenetic tree (Fig. S1). By comparing the genetic compositions of 14 selected strains, we found the insertion of a putative glycosyltransferase gene, AM305_00150, in the *A. minor* NM305 genome (Fig. 1*B*). The protein encoded by the AM305_00150 gene was predicted to have a GT-B fold and classified into the UDP-glycosyltransferase/glycogen phosphorylase superfamily in the SUPFAM database (13). Homologous proteins were searched by Blastp in the BLAST nonredundant protein sequence database. The top-ranked hits were the GT-B fold glycosyltransferases Ag1Agt (*Actinobacillus genomosp.* 1), ApAgt (*A. pleuropneumoniae*), AlAgt (*Actinobacillus lignieresii*), OOF46308.1 (*Rodentibacter trehalosifermentans*), and WP_044250115.1 (*Kingella negevensis*) with 53%, 51%, 51%, 45%, and 41% sequence identities and 93%, 93%, 93%, 91%, and 91% query coverages, respectively (Fig. S2). Next, we searched for structural homologs in the PDB database, but no significant sequences with known structures were found.

The genes encoding ApNGT and ApAgt form an operon, RimO-ApNGT-ApAgt, in the *App* genome (Fig. 1*C*). The NGT gene is well conserved in the RimO operon among species. By contrast, the Agt gene is limited to *A. pleuropneumoniae*, *A. lignieresii*, and *A. genomosp. 1.* Interestingly, the AM305_00150 gene is homologous to the Agt genes, but resides in another operon containing rsmB in the *A. minor* genome (Fig. 1*B*). It is reasonable to assume that the AM305_00150 gene was incorporated within the *A. minor* genome by a horizontal gene transfer after the between-species divergence, because no glycosyltransferase genes were detected in the corresponding regions in the genomes of the *A. minor* 202 and *A. minor* isolate SUG1863 strains (Fig. 1*B*). Thus, we expected that the AmGGT protein encoded by the AM305_00150 gene would have unique enzymatic properties.

### Glycosyltransfer activity of the AmGGT protein

We expressed the AmGGT protein as a recombinant protein with a His_6_-tag at the N terminus, using *Escherichia coli* cells as the host. The recombinant protein was purified with a three-step protocol of metal affinity, ion-exchange, and gel-filtration chromatography. Considering that the ApAgt protein uses glucose-conjugated peptides as acceptor molecules (8), we designed an acceptor glycopeptide, Ac-AAYN(Glc)VTKR(K-TAMRA), and named it Glc_1_-peptide. The asparagine residue in the glycosylation consensus, Asn-Val-Thr, was modified with a glucose residue by the ApNGT protein. The N-terminal α-amino group of the peptide was blocked with an acetyl group and the side chain ε-amino group of the C-terminal lysine residue was modified with a fluorescent TAMRA group. The catalytic activity of the AmGGT protein was assayed with UDP-Glc as the donor and the Glc_1_-peptide as the acceptor (Fig. 1*D*). Normal-phase ultrahigh performance liquid chromatography (UPLC) was used to separate and monitor the glucose transfer to the mono- and oligo-glucose chains on the Glc_1_-peptide. In the chromatogram, an increasing number of glucose residues attached to the Glc_1_-peptide results in a later elution time. The ApAgt protein repeatedly transferred a glucose residue as expected, whereas the AmGGT protein did not transfer any glucose residues to the Glc_1_-peptide substrate (Fig. 1*D*).

Next, we tested an oligosaccharide as a substitute for the glycopeptide substrate, considering that some protein glycosyltransferases only use oligosaccharides as acceptor substrates. We labeled isomaltose (Glcα1-6Glc) with 2-aminopyridine (2-AP) for fluorescent detection. Pyridyl amination (PA) involves ring-opening at the reducing-end monosaccharide residue (Fig. S3). In the following text, the PA derivative of isomaltose is referred to as Glcα1-6Glc-PA. The AmGGT and ApAgt enzymes both used Glcα1-6Glc-PA as an acceptor (Fig. 1*E*). It is noteworthy that the AmGGT protein repeatedly transferred a glucose residue much more efficiently than the Agt protein to the oligo-glucose-PAs. The oligo-glucose chains thus produced consisted of two to eight glucose residues.

**Figure 2:**
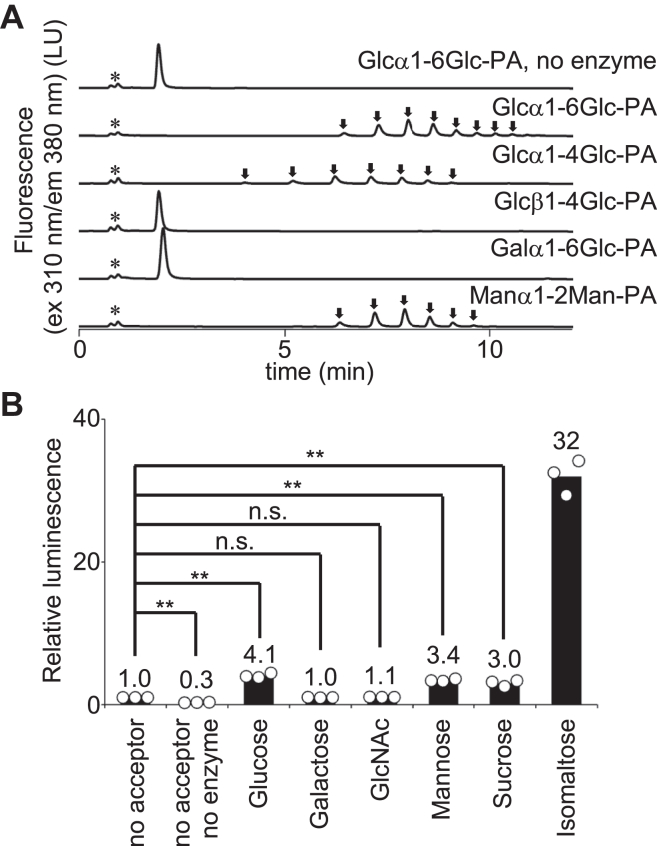
**Oligosaccharide acceptor specificity of the AmGGT protein.***A*, the catalytic activity of AmGGT was measured using various PA-labeled disaccharides as acceptors. The UPLC conditions were the same as in Figure 1*E*. *Asterisks* indicate the positions of nonspecific peaks. *Arrows* indicate the enzymatic reaction products. *B*, the UDP-Glc hydrolysis activity was measured using the UDP-Glo method, which converts UDP to ATP and measures the luminescence produced by the luciferase reaction. Monosaccharides (Glc, Gal, GlcNAc, Man), sucrose, and isomaltose were used as acceptor molecules. The luminescence intensity was normalized to that measured without acceptor molecules (no acceptor). The *circles* represent the data points obtained from three independent experiments. The bar heights indicate the mean values. Statistical analyses were conducted by one-way ANOVA followed by Dunnett's two-sided *post hoc* test. ∗∗*p* < 0.001 and n.s.: nonsignificant. AmGGT, *Actinobacillus minor* glucoside-glucosyltransferase; PA, pyridyl amination; UPLC, ultrahigh performance liquid chromatography.

To investigate the specificity of oligosaccharides as acceptor substrates, maltose (Glcα1-4Glc), cellobiose (Glcβ1-4Glc), melibiose (Galα1-6Glc), and mannobiose (Manα1-2Man) were labeled with 2-AP and tested as acceptor substrates. The chemical structures of these labeled disaccharides are shown in the (Fig. S3). Glcα1-4Glc-PA and Manα1-2Man-PA served as acceptors in addition to Glcα1-6Glc-PA, demonstrating that glucose and mannose residues at the nonreducing end with an α-anomeric configuration are the acceptors for the AmGGT protein (Fig. 2*A*). Note that maltose (Glcα1-4Glc) generates Glcα1-4Glc-PA, whose linker structure differs from the Glcα1-6Glc-PA generated from isomaltose (Glcα1-6Glc). Glcα1-4Glc-PA was compatible with Glcα1-6Glc-PA as an acceptor for the AmGGT protein. Glcβ1-4Glc-PA and Galα1-6Glc-PA were not accepted as substrates, suggesting that the glucose residue with the β-anomeric configuration and the galactose residue at the nonreducing end are both inactive as acceptors.

To assess the possible side effects of the fluorescent labeling of acceptors on the catalytic activity of AmGGT, the UDP-Glc donor hydrolysis was measured in the presence of various monosaccharide and disaccharide as acceptors (Fig. 2*B*). Note that these acceptors do not contain fluorescent PA groups. The amounts of released UDP indicated that glucose and mannose increased the hydrolysis of UDP-Glc by the AmGGT protein, whereas galactose and GlcNAc were inactive. These results demonstrated that the PA labeling of acceptors at the reducing end has minimal unwanted effects on the acceptability of the nonreducing-end monosaccharide residues.

The disaccharide isomaltose (Glcα1-6Glc) is an acceptor substrate with 10-fold higher hydrolytic activity than the monosaccharide glucose. Sucrose (Glcα1-2Fru), in which a glycosidic bond is formed between the reducing ends of glucose and fructose, was comparable in activity to glucose and mannose, indicating that the residues next to the nonreducing end modulate the catalytic activity of the AmGGT protein. The UDP-Glo assay was then used to study the effects of oligosaccharide chain lengths from Glc_2_ to Glc_5_ on UDP release (Fig. S4). The AmGGT protein primarily preferred Glc_3_ (isomaltotriose) as an acceptor.

### Donor specificity of the AmGGT protein

**Figure 3:**
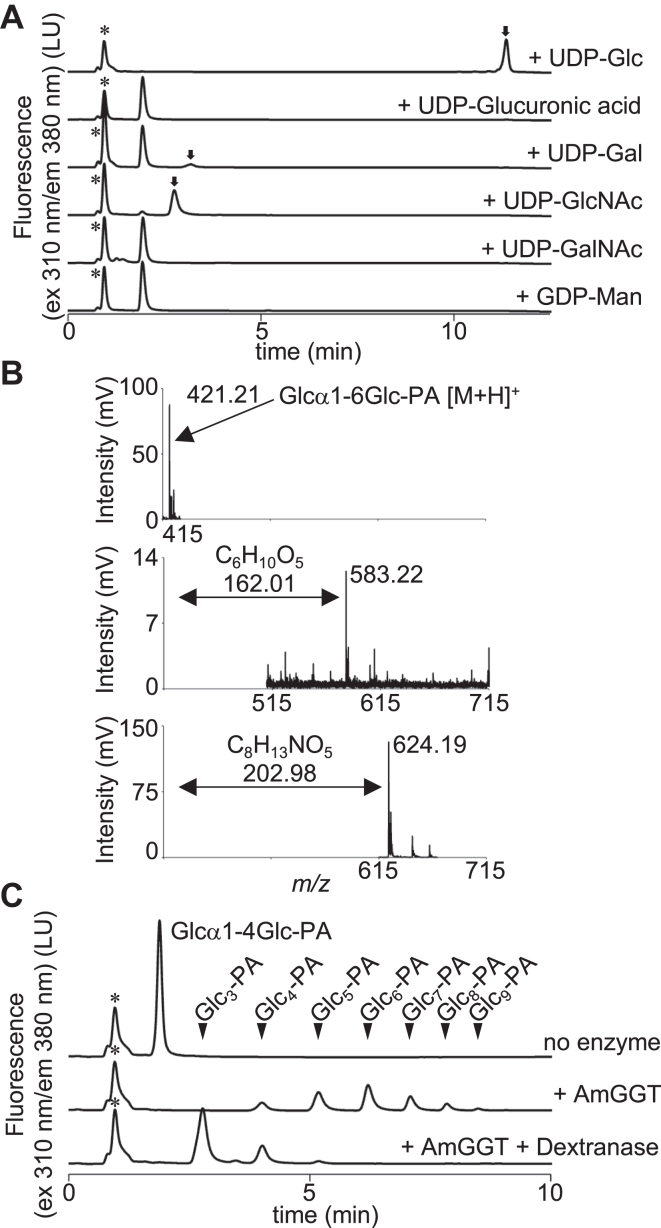
**AmGGT activity in the presence of various nucleotide sugars as donor substrates.***A*, the AmGGT protein was incubated with nucleotide sugars and Glcα1-6Glc-PA as the acceptor for 16 h to detect poor reactions. The reaction products were separated by normal-phase chromatography. *Asterisks* indicate the positions of nonspecific peaks. *Arrows* indicate the enzymatic reaction products. *B*, MALDI-TOF MS spectra in the positive mode, using DHB as the matrix. Glcα1-6Glc-PA as a control (*top*), and the AmGGT reaction products with UDP-Gal (*middle*), and UDP-GlcNAc (*bottom*). The *m*/*z* values of the observed peaks are indicated. The *double-headed arrows* indicate the differences in the *m/z* values from the Glcα1-6Glc-PA peak. *C*, dextranase digestion of the AmGGT products. The oligo-glucose chain was generated by AmGGT using PA-labeled maltose (Glcα1-4Glc-PA) as the acceptor and UDP-Glc as the donor before the dextranase treatment. The *triangles* indicate the elution positions of the oligo-glucose-PA molecules as markers. AmGGT, *Actinobacillus minor* glucoside-glucosyltransferase; PA, pyridyl amination.

The ApAgt protein reportedly has high specificity for UDP-Glc as a donor molecule (8). We tested six nucleotide sugar molecules, UDP-Glc, UDP-GlcA (glucuronic acid), UDP-GlcNAc, UDP-Gal, UDP-GalNAc, and GDP-Man, to assess the donor specificity of the AmGGT protein. The AmGGT protein used UDP-Glc, UDP-Gal, and UDP-GlcNAc as donor substrates, but not UDP-GlcA, UDP-GalNAc, or GDP-Man (Fig. 3*A*). The reaction products were recovered from the eluents and analyzed by matrix-assisted laser desorption/ionization time-of-flight mass spectrometry (MALDI-TOF-MS) (Fig. 3*B*). These analyses revealed that the *m*/*z* values of the reaction products in the presence of UDP-Gal and UDP-GlcNAc were consistent with those calculated assuming the addition of a galactose residue (*m*/*z* 583.22 [M+H]^+^) and a GlcNAc residue (*m*/*z* 624.19 [M+H]^+^) to the Glcα1-6Glc-PA molecule. The sequential additions of monosaccharide residues occurred with UDP-Glc as the donor substrate but were not observed with UDP-Gal or UDP-GlcNAc (Fig. 3*A*), because a Gal residue (and probably a GlcNAc residue) at the nonreducing end is not a suitable acceptor (Fig. 2*A*). The AmGGT catalytic reaction product (Glc_2_-Glcα1-6Glc-PA) was eluted at the same retention time (about 4 min) as one of the ApAgt products in the UPLC chromatograms (+Glc_2_, Fig. 1*E*). The identical elution times advocate that the AmGGT protein transfers glucose to the acceptors *via* an α-1,6-glycosidic linkage. To further confirm the type of linkage in the elongated glucose chain, an endodextranase, which specifically hydrolyzes the α-1,6-linked glycosidic linkage, was added to the reaction mixture after the AmGGT enzymatic reaction (Fig. 3*C*). The shorter retention times of the product peaks after the endodextranase digestion indicated the shortening of a carbohydrate chain, suggesting that the AmGGT protein catalyzed the formation of the α-1,6-glycosidic bonds. Ultimately, the linkage type of the glycosidic bonds must be determined using NMR. This result supports the proposal that the AmGGT protein transfers the glucose moiety from UDP-α-D-Glc with the retention of the anomeric configuration, and thus classifies AmGGT as a member of the retaining GT-B fold glycosyltransferase family.

### Crystal structure of the AmGGT protein

**Figure 4:**
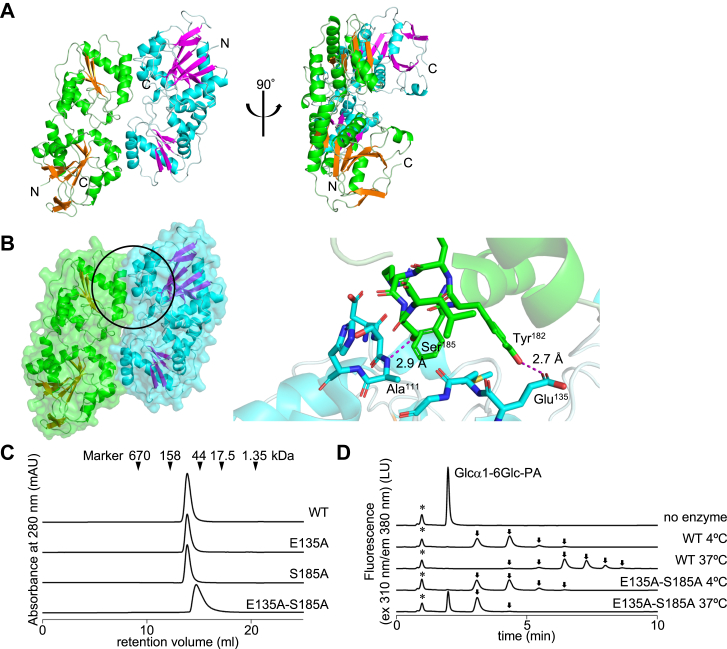
**Crystal structure of the AmGGT protein and catalytic activities of its mutants generated based on the dimeric homodimer model.***A*, homodimeric structure reconstituted from a single molecule of the AmGGT protein in the asymmetric unit. The second subunit was generated according to the crystal lattice symmetry using the PyMOL program. Another view is shown after rotating 90 degrees. *B*, molecular surface and the close-up view of the interface between the two subunits. In the enlarged view of the *circled area* (*left*), the amino acid residues at the interface are shown as *stick models* (*right*). *C*, gel-filtration chromatograms of WT AmGGT and its mutants. The *triangles* indicate the positions of protein size markers. *D*, effects of temperature on the catalytic activities of the WT and E135A-S185A mutant proteins. The reaction mixtures were incubated at 4 °C and 37 °C. *Asterisks* indicate the positions of nonspecific peaks. *Arrows* indicate the enzymatic reaction products. AmGGT, *Actinobacillus minor* glucoside-glucosyltransferase.

Initial crystallization screening yielded small crystals, which were used as microseeds to generate crystals of appropriate size for X-ray diffraction measurements. The crystal structure of the AmGGT protein was determined to the resolution of 1.85 Å (Fig. 4*A* and Table 1). Although the AmGGT protein was mixed with isomaltose (as an acceptor), no density corresponding to the isomaltose molecule was present in the electron density map, presumably due to the weak affinity of isomaltose for the AmGGT protein. The AmGGT protein consists of two lobe domains, which are typically found in the GT-B fold proteins. A substrate-binding groove is formed at the interface between the two lobe domains. Although the asymmetric unit contained one molecule of the AmGGT protein, the gel-filtration chromatography suggested that the AmGGT protein exists as a homodimer in solution (Fig. 4*C*). A dimeric structure was generated according to the 2-fold rotational crystal lattice symmetry (Fig. 4*A*). To confirm the homodimer formation and estimate the arrangement of the two subunits, we introduced mutations at the interface between the two subunits (Fig. 4*B*). Three mutant proteins, E135A, S185A, and E135A-S185A, were generated and analyzed by gel-filtration chromatography (Fig. 4*C*). The two single mutants, E135A and S185A, each eluted at the same retention volume as the WT AmGGT protein. By contrast, the double mutant, E135A-S185A, eluted at a higher retention time and showed a broader peak shape than the WT protein. The molecular masses were estimated to be 80 kDa for the WT protein and 36 kDa for the E135A-S185A mutant protein. To confirm the integrity of the E135A-S185A mutant protein, eluted peaks were collected and subjected to SDS-PAGE analysis under nonreducing conditions (Fig. S5). No protein degradation was observed with the E135A-S185A mutant protein. In sum, the AmGGT protein is a homodimer with 2-fold rotational symmetry in solution (Fig. 4*A*).

**Table 1:** Data collection and refinement statistics^a^

Data collection	Apo form	UDP complex
Diffraction source	SPring-8 BL45XU	SLS X06SA
Wavelength (Å)	1.00000	1.00003
Resolution range (Å)	46.44–1.85 (1.89–1.85)	42.52–1.80 (1.84–1.80)
Space group	*C*2	*C*2
a, b, c (Å)	93.592, 44.312, 90.754	100.136, 43.390, 95.823
α, β, γ (°)	90.000, 113.745, 90.000	90.000, 117.440, 90.000
Reflections (measured/unique)	55,944/29,001	229,704/32,975
Completeness (%)	98.9 (87.5)	96.7 (96.5)
Mean I/σ(I)	38.7 (17.2)	33.0 (7.2)
Multiplicity	1.9 (1.9)	7 (6.7)
*R*_pim_^b^	0.020 (0.099)	0.013 (0.088)
CC_1/2_	0.998 (0.885)	1.000 (0.989)
Wilson B-factor (Å^2^)	7.43	21.92
Refinement statistics		
Resolution range (Å)	46.49–1.85	42.56–1.80
*R*_work_/*R*_free_ (%/%)^c^	15.6/21.1	15.8/20.1
No. of nonhydrogen atoms		
Protein	2779	2796
Ligand	0	29
Water	203	215
Average B factor (Å^2^)	11.6	28.2
Rms deviations from ideal^d^		
Bond lengths (Å)	0.008	0.008
Bond angles (°)	1.47	1.45
Ramachandran plot, residues in (%)		
Most favorable region	97	97
Additional allowed region	3	3
PDB entry	8XSH	8XSG

a Values in parentheses are for the highest resolution shell.

b *R*_pim_ = $\sum_{hkl}\sqrt{1/n-1}\;\sum_{i=1}^{n}\left|I_{i}\left(hkl\right)-\bar{I}\left(hkl\right)\right|\;/\;\sum_{hkl}\sum_{i=1}^{n}I_{i}\left(hkl\right)$.

c *R*_work_/*R*_free_ = ∑|Fo—Fc|/∑|Fo|. *R*_work_ was calculated from the working set (95% of the total reflections). *R*_free_ was calculated from the test set (5% of the total reflections). The test set was not used in the refinement.

d Rms, root mean square.

We measured the enzymatic activities of the WT and E135A-S185A mutant proteins to examine the effects of dimerization on the catalytic activity (Fig. 4*D*). At 4 °C, the two proteins showed similar glucosyl transfer activities from UDP-Glc to Glcα1-6Glc-PA. In contrast, at 37 °C, the catalytic activity of the WT protein increased, but that of the E135A-S185A mutant protein decreased. These results suggest that the homodimeric structure of the AmGGT protein contributes to its thermal stability under physiological temperature conditions in the porcine host.

### Mechanism of donor recognition

**Figure 5:**
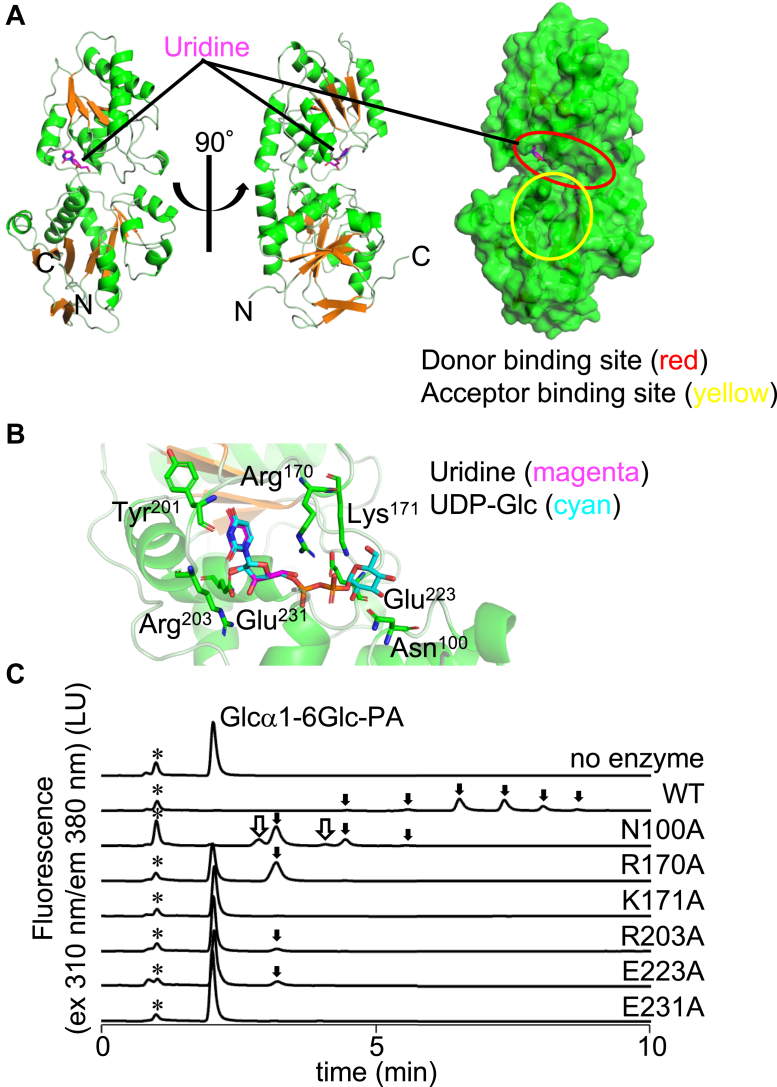
**Crystal structure of the AmGGT–UDP complex and catalytic activities of its mutants generated based on the UDP-Glc docking model.***A*, crystal structure and the molecular surface of the AmGGT–UDP complex. For clarity, only one of the two subunits is shown. The uridine moiety of the bound UDP (*magenta*) is drawn with a *stick model*. The *red circle* indicates the location of the donor-binding site, and the *yellow circle* indicates the location of the acceptor-binding site. *B*, close-up view of the donor-binding site. The modeled UDP-Glc molecule (*cyan*) was superimposed on the uridine part of the bound UDP (*magenta*). Key residues within 3.2 Å of the modeled UDP-Glc molecule are shown as *stick models* with residue numbers. *C*, AmGGT mutants were generated by mutating the key residues involved in donor substrate recognition, and their glucosyl transfer activities were measured. The *black arrows* indicate common α-1,6-glycosidic bond products, identical to those observed in the WT. The *white arrows* indicate new product peaks specific to the N100A mutant. *Asterisks* indicate the positions of non-specific peaks. AmGGT, *Actinobacillus minor* glucoside-glucosyltransferase.

To study the donor substrate recognition, the WT AmGGT protein was premixed with UDP before crystallization. The crystal structure was determined to the resolution of 1.80 Å (Fig. 5*A*, and Table 1). The uridine moiety was visible in the electron density map, but the electron density of the diphosphate group, particularly the density for the β-phosphate of the UDP molecule was ambiguous. Although the electron density for the α-phosphate was better than that for the β-phosphate, its position could not be determined with certainty. The bound uridine moiety contacts the side chain atoms of three amino acid residues (Arg^170^, Arg^203^, and Glu^231^) and the main chain atoms of Tyr^201^. We docked a UDP-Glc molecule into the AmGGT–UDP complex structure by superimposing the uridine part of UDP as a guide using the PyMOL program (http://www.pymol.org/) (Fig. 5*B*). The atomic coordinates of UDP-Glc (Chemical ID: UPG) was taken from PDB. The C-6 carbon of the glucose residue was moved close to the presumed acceptor-binding site manually while avoiding atomic collisions. In the docking structure, the side chain atoms of Asn^100^, Lys^171^, and Glu^223^ are involved in the recognition of the glucose moiety of UDP-Glc and appeared necessary for proper acceptor substrate positioning. Next, to evaluate the docking model, we generated alanine mutants and measured their glucosyl transfer activities to Glcα1-6Glc-PA (Fig. 5*C*). All the mutants, and especially, four mutants, K171A, R203A, E223A, and E231A, had lower glucosyl transfer activity than the WT protein. In the case of N100A, in addition to the peaks common to the WT AmGGT products (black arrows), new product peaks were observed (white arrows), suggesting that the newly added glucose residues are linked *via* a non-α-1,6 glycosidic bond to the nonreducing end or a hydroxyl group in the structure other than the nonreducing end glucose residue.

No metal ion density was detected in the electron density map. We thus examined the requirement for metal ions for the glucosyl transfer activity (Fig. S6*A*). The addition of CoCl_2_, NiCl_2_, and ZnSO_4_ decreased the catalytic activity, but MgCl_2_, CaCl_2_, and MnCl_2_ had no effect. In addition, EDTA, a metal cation chelating agent, did not affect the catalytic activity. These results demonstrated that AmGGT is a metal ion-independent glycosyltransferase and are consistent with the fact that the GT-B fold enzymes are generally metal-independent (14). The AmGGT protein was incubated with UDP-Glc and Glcα1-6Glc-PA at pH 4.0 to 10.0. The AmGGT activity was optimal at neutral to alkaline pH (Fig. S6*B*).

### Mechanism of acceptor recognition

**Figure 6:**
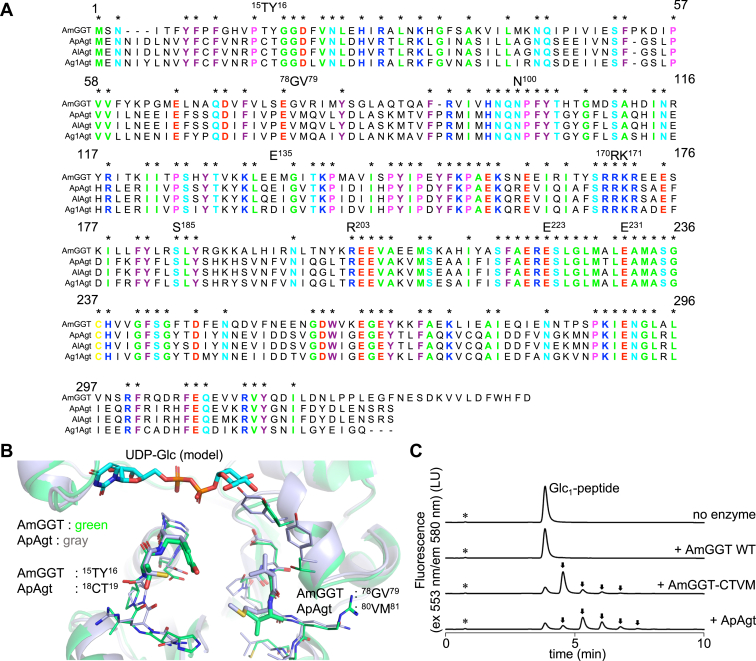
**Comparison of the structural basis of the acceptor recognition between the AmGGT and ApAgt proteins.***A*, multiple sequence alignment of the AmGGT and Agt proteins. The *asterisks* and *colored fonts* indicate the conserved amino acid residues. The key amino acid residues focused on in this study are marked above the alignment and numbered according to the AmGGT sequence. *B*, close-up view of the acceptor-binding site in the AmGGT structure (*green*) in complex with a UDP-Glc model (*cyan*). The AlphaFold2 predicted structure of the ApAgt protein was superimposed (*gray*). Amino acid residues of interest are shown as *thick stick models*. *C*, conversion of the acceptor specificity of the quadruple mutant (AmGGT-CTVM) from oligosaccharides to glycopeptides. The catalytic activity of the mutant was measured using UDP-Glc as the donor and Glc_1_-peptide as the acceptor. *Arrows* indicate the enzymatic reaction products. AmGGT, *Actinobacillus minor* glucoside-glucosyltransferase.

A multiple sequence alignment revealed that the amino acid residues forming the UDP-Glc binding site are conserved between AmGGT, ApAgt, and the homologous proteins (Fig. 6*A*). The structure of the AmGGT protein was superimposed on an AlphaFold2 predicted structure of the ApAgt protein (Fig. 6*B*). However, the amino acid residues that constitute the acceptor recognition site were not conserved between the AmGGT and ApAgt proteins. The pairs of Thr^15^ and Tyr^16^, and Gly^78^ and Val^79^ in the AmGGT protein correspond to Cys^18^ and Thr^19^, and Val^80^ and Met^81^ in the ApAgt protein, respectively. The multiple sequence alignment confirmed species-specific amino acid variations at residues 15 to 16 and 78 to 79, according to the numbering of the AmGGT sequence (Fig. 6*A*). We hypothesized that the amino acid residues 15 to 16 and 78 to 79 determine the acceptor substrate specificity of the AmGGT protein and designed the quadruple mutant (AmGGT-CTVM) by replacing the four amino acid residues (Thr^15^, Tyr^16^, Gly^78^, and Val^79^) with the corresponding amino acid residues (Cys^18^, Thr^19^, Val^80^, Met^81^) in the ApAgt protein. We measured the glucosyl transfer activity using the Glc_1_-peptide as the acceptor. As expected, the AmGGT-CTVM mutant acquired the glucose transfer activity to the glycopeptide substrate (Fig. 6*C*). We speculated that the quadruple mutant also transfers glucose *via* an α-1,6-glycosidic linkage to the Glc_1_-peptide. The catalytic product of AmGGT-CTVM eluted at the same retention time as the ApAgt reaction product when the Glc_1_-peptide was used as the acceptor (Fig. 6*C*). Thus, it is reasonable to conclude that the quadruple mutant transfers glucoses to the Glc_1_-peptide *via* an α-1,6-glycosidic linkage.

### Branched glycans as acceptor molecules

**Figure 7:**
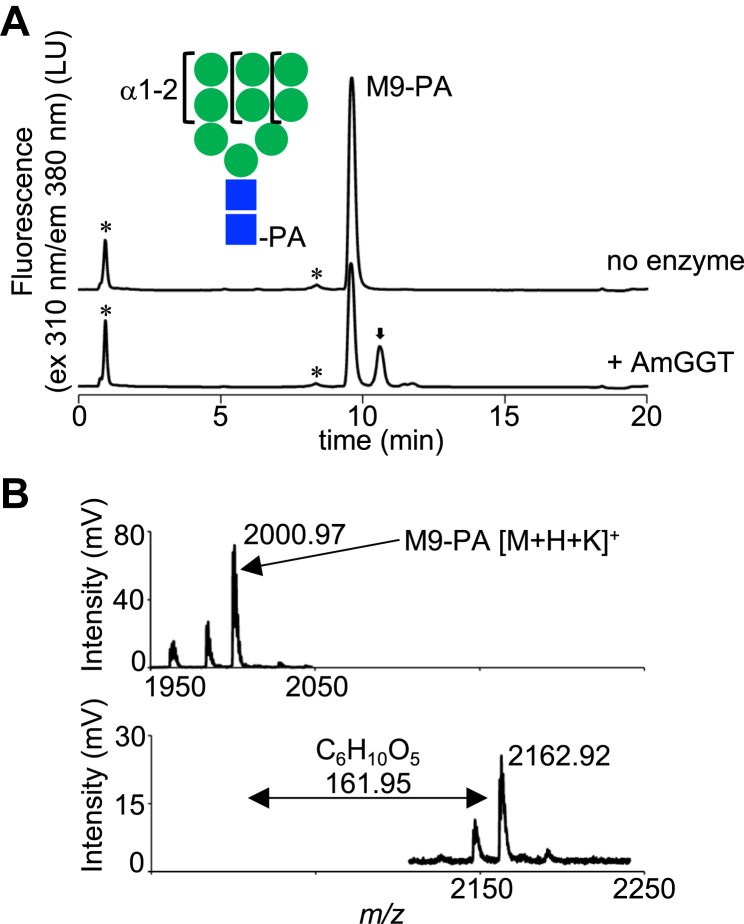
**AmGGT can modify branched oligosaccharides.***A*, glucosyl transfer activity of WT AmGGT protein to a eukaryotic high mannose-type *N*-glycan (M9-PA) as the acceptor. UDP-Glc was used as the donor. *Asterisks* indicate positions of nonspecific peaks. The *arrow* indicates the reaction product. The M9 *glycan* structure is shown in the *inset*. The *blue squares* represent GlcNAc, and the *green circles* represent Man. *B*, MALDI-TOF MS spectra in the positive ion mode, using DHB as the matrix. M9-PA was detected in the peak collected from the reaction mixture before the reaction (*top*), and Glc-M9-PA was detected after the incubation (*bottom*). The *m*/*z* values of the observed peaks are indicated. The *double-headed arrow* indicates the difference in the *m/z* value from the M9-PA peak. AmGGT, *Actinobacillus minor* glucoside-glucosyltransferase; PA, pyridyl amination.

Because the acceptor-binding site of the AmGGT protein is wide-open (Fig. 5*A*), we hypothesized that branched glycans may be available as acceptor substrates. To explore the biological functions and applications of the AmGGT protein, we tested branched glycans as acceptor substrates. The eukaryotic high-mannose type *N*-glycans contain mannose residues with α-1,2-linkages in the three branched arms. The same structure exists in mannobiose (Manα1-2Man), which was a good acceptor substrate for the AmGGT protein (Fig. 2*A*). Thus, we tested whether *N*-glycan M9 (Man_9_GlcNAc_2_, which contains three terminal Manα1-2Man structures) served as an acceptor. New peaks were observed in the UPLC chromatogram when the AmGGT protein was added to the reaction mixture (Fig. 7*A*). The largest new peak was collected and analyzed by MALDI-TOF-MS (Fig. 7*B*). The *m*/*z* value of 2162.92 is consistent with the addition of a glucose residue to the M9-PA molecule (calculated *m*/*z* 2162.72 [M+H+K]^+^). The *N*-glycan G3M9 (Glc_3_Man_9_GlcNAc_2_, which contains two terminal Manα1-2Man structures) also served as an acceptor (Fig. S7). These results indicate that the AmGGT protein can modify branched oligosaccharides, as well as linear oligosaccharides.

## Discussion

Enzymatic synthesis has advantages in stereoselectivity and regioselectivity over organic synthesis, which requires several protection/deprotection steps (15). Advances in genome informatics have led to the discoveries of secondary metabolites produced by bacteria and the identification of their synthetic pathways (16). Researchers have recently discovered useful glycosyltransferases in the genomes of nonmodel organisms. The detailed characterization of putative glycosyltransferases is an obligatory step for producing drug leads, cosmetics, nutrients, and sweeteners by aglycone glycosylation through metabolic engineering (3, 17). Although these glycosyltransferase candidates are annotated based on sequence conservations and operon structures, the enzymatic activities for most putative glycosyltransferases have not been verified.

In this study, we analyzed the protein (AmGGT) encoded by the AM305_00150 gene in the *A. minor* NM305 strain. Despite high sequence identity (>50%) with the *A. pleuropneumoniae* Agt protein (ApAgt), the acceptor substrate specificity of AmGGT is distinct: Unlike ApAgt, AmGGT cannot use glycopeptides as acceptor substrates (Fig. 1*D*). Instead, the AmGGT protein uses mono- and oligo-glucose chains as acceptors (Fig. 2*B*). Furthermore, AmGGT elongates an oligosaccharide chain efficiently by repeatedly adding a glucose residue to the nonreducing end of glucose chains (Fig. 1*E*). In contrast, the ApAgt protein transfers only one or at most two glucose residues. The AmGGT protein can transfer glucose residues to branched eukaryotic high-mannose *N*-glycans (Figs. 7*A* and S7). To date, only autotransporter adhesion proteins have been identified as substrates of the ApAgt protein (18). The unique substrate specificity of AmGGT suggests that the biological role of AmGGT in *A. minor* is different from that of ApAgt in *A. pleuropneumoniae.* Further studies to identify the natural substrates of AmGGT are needed to understand the biological role of AmGGT in *A. minor*.

The binding site of the donor molecule was identified in the crystal structure, with the aid of model building (Fig. 5*B*). The AmGGT protein prefers UDP-Glc as a donor but can also use UDP-Gal and UDP-GlcNAc, albeit with lower efficiency (Fig. 3*A*). A mutational analysis suggested that Asn^100^ could be involved in regulating the regioselective formation of the α-1,6-glycosidic bond (Fig. 5*C*). In this study, we did not determine the glycosidic linkage types of the catalytic products, except for the estimation by endodextranase digestion. For different donor and acceptor substrates, the type of glycosidic bond must be determined by NMR spectroscopy in future studies. By reference to the three-dimensional structures, we inferred that the Thr^15^-Tyr^16^ and Gly^78^-Val^79^ residues contribute to the specificity for oligosaccharides as acceptor molecules (Fig. 6*B*). In the AmGGT structure, the side chain of Tyr^16^ protrudes into the substrate-binding groove, narrowing the acceptor-binding site. This bulged structure likely prevents the binding of glycopeptide substrates, such as the Glc_1_-peptide, to the acceptor-binding site. Presumably, the substrate-binding groove of the AmGGT protein interacts with multiple parts of acceptor molecules. Indeed, the AmGGT protein utilized Glc_n_ (n = 2–5) (from isomaltose to isomaltopentaose without PA labeling) as better acceptors than the monosaccharide glucose (Figs. 2*B* and S4). The optimal length was Glc_3_ (isomaltotriose), suggesting that the size of the acceptor-binding site can accommodate three monosaccharide residues. Because the Thr^15^-Tyr^16^ and Gly^78^-Val^79^ residues are unique to *A. minor* (Fig. 6*A*), the AmGGT protein may have different intracellular functions from the Agt proteins.

The pleuropneumonia caused by *App* ranks among the top 10 infectious diseases reported in swine, and its spread and high mortality rates have significant economic impacts (6, 19). In recent years, resistance to existing antibiotics has been reported in some countries, and thus the needs for vaccine development and new drug target molecules are more urgent than ever (20, 21). Recombinant glycosylated toxin proteins are antigen candidates for *App* vaccine research (22). Unfortunately, except for *App*, the details of the glycosylations on adhesin proteins in the *Actinobacillus* genus and closely related genera are unknown. In this study, we showed that proteins homologous to ApAgt are conserved among at least three species, including *App*, based on the phylogenetic analysis and the comparison of the operon structures (Figs. 1*B* and S1). The AM305_00150 gene that encodes the AmGGT protein is in a different operon from the Agt encoding genes. As expected, the AmGGT protein has a distinct specificity for acceptor molecules from the ApAgt protein, despite the high amino acid similarity to the ApAgt proteins: The AmGGT protein cannot use glycopeptides as an acceptor (Fig. 1*D*), but uses oligosaccharides (Fig. 1*E*). This suggests that the sequential glucosylation reaction to the adhesin proteins does not occur in the cytoplasm of *A. minor* cells. The crystal structures of the AmGGT protein will be useful as a model for Agt protein design and molecular dynamics simulations of interactions with substrates and inhibitors (Figs. 4 and 5). The altered acceptor specificity of the AmGGT protein due to the quadruple mutation in the acceptor-binding site provides useful clues for the future biotechnological applications of the AmGGT and Agt proteins (Fig. 6*C*).

The sequential glucosylation reaction and unique substrate specificity of AmGGT are useful in applied research areas for the generation and modification of new glycosylated molecules. The repeated α-1,6-linked oligo-glucose structure is also present in the O-antigen of *Helicobacter pylori* (23). An α-1,6-glucosyltransferase (HP0159) was used as an antigen to prepare an antibody against the α-1,6-glucan epitope of *H. pylori* lipopolysaccharide, which was effective in mice (23).

Last, the BLAST search using the AmGGT sequence as a query revealed several proteins with high amino acid sequence homology in bacteria other than the *Actinobacillus* genus: OOF46308.1 (*R. trehalosifermentans*) and WP_044250115.1 (*K. negevensis*) with 45% and 41% sequence identities, respectively. The biological functions of these proteins are unknown. We expect that the present study will be helpful for future studies on these putative glucosyltransferase proteins.

## Experimental procedures

### Reagents

Nucleotide sugars and acceptor oligosaccharides were purchased as follows: UDP-Glc (SIGMA U4625), UDP-GlcNAc (CAY 20353), UDP-Gal (CAY 34664), UDP-GalNAc (CAY 36506), GDP-Man (TOYOBO RYM7210), isomaltose (TCI I0321), maltose (Nacalai 21115-15), 2α-mannobiose (SIGMA M1050), cellobiose (TCI C0056), melibiose (TCI M0050), isomaltotriose (TCI I0329), isomaltotetraose (TCI I0855), and isomaltopentaose (TCI I0854), and dissolved in water. 2-AP was purchased from WAKO (011-14181).

### DNA sequences

The 16S rRNA sequences of *the Actinobacillus* genus were retrieved from the NCBI. Multiple sequence alignment was performed with the MAFFT program (24), and the resultant alignment was used for the subsequent phylogenetic analysis. The phylogenetic tree was generated using the Archaeopteryx.js web software (25), which employs the neighbor-joining method. The genetic organizations around the rsmB and rimO gene loci in the *Actinobacillus* genus genomes were obtained from the NCBI.

### Protein production

The ORF sequences, AmGGT (AM305_00150) and ApAgt (APP7_1696), were retrieved from the NCBI database. The ORF DNAs were chemically synthesized (Genewiz) and amplified by PCR. The PCR products were cloned into the pET47b vector with an N-terminal hexa-histidine tag followed by the 3C protease site. The inserted ORFs were validated by DNA sequencing. The AmGGT and ApAgt proteins were expressed as His-tagged proteins in *E. coli* strain BL21(DE3) cells. The transformed *E. coli* cells were grown in ZYM-5052 autoinduction medium at 37 °C for 4 h and then at 20 °C for 16 h (26). The cells were harvested by centrifugation at 5000*g* at 4 °C for 10 min and resuspended in suspension buffer (20 mM Tris–HCl, pH 8.0, 250 mM NaCl) containing 20 mM imidazole. After disruption by sonication and centrifugation at 8000*g* at 4 °C for 10 min to remove debris, the supernatant was centrifuged at 50,000*g* at 4 °C for 30 min. The supernatant was loaded onto a Ni-Sepharose column (GE Healthcare), which was pre-equilibrated with suspension buffer containing 20 mM imidazole. The column was then washed with suspension buffer containing 40 mM imidazole. The His-tagged proteins were eluted with suspension buffer containing 200 mM imidazole. The His-tag was cleaved by an overnight incubation with 3C protease.

For the biochemical assay, the 3C protease digestion mixtures were further separated by gel-filtration chromatography, using a Superdex 200 Increase 10/300 column (GE Healthcare) equilibrated in buffer (50 mM Tris–HCl, pH 7.4, 150 mM NaCl, 10% (w/v) glycerol). The peak fractions were collected, frozen in liquid nitrogen, and stored at −80 °C until use.

### Crystallization

After diluting the 3C protease digestion mixture 5-fold with 20 mM Tris–HCl, pH 8.0, the diluted mixture was loaded on a HiTrapQ column (GE Healthcare). Solvent A was 20 mM Tris–HCl, pH 8.0, and 50 mM NaCl, and solvent B was 20 mM Tris–HCl, pH 8.0, and 1 M NaCl. A linear gradient of solvent B was applied from 0% B to 40% B. The peak fractions were pooled and concentrated using Amicon Ultra concentrators (Millipore) with a molecular mass cutoff of 10 kDa. The concentrated proteins were further purified by gel-filtration chromatography using a Superdex 200 Increase 10/300 column (GE Healthcare) in buffer (50 mM Hepes, pH 7.4, 200 mM NaCl, 0.1 mM EDTA, and 5% (w/v) glycerol). The peak fractions were pooled and concentrated to 12 mg/ml. Before crystallization, the protein solution was mixed with isomaltose or UDP (CAY 18137) at a final concentration of 3 mM and incubated at 25 °C for 30 min. The crystallization screening was performed using the sitting-drop vapor-diffusion method at 20 °C. Crystals appeared after 3 days from drops consisting of 1.5 μl of protein solution and 1.5 μl of reservoir solutions containing 20% (w/v) PEG3350. Crystallization was optimized by microseeding. The mixture of 1.5 μl of protein solution, 0.2 μl of seed solution, and 1.5 μl of reservoir solution (0.1 M MES, pH 6.5, 0.2 M sodium formate, 14% (w/v) PEG3350) was subjected to the hanging-drop vapor-diffusion method at 20 °C.

### X-ray diffraction measurements and data processing

Crystals were cryo-protected with a cryo-protection solution, 0.1 M MES, pH 6.5, 0.2 M sodium formate, 23% (w/v) PEG3350, and 4% (w/v) PEG400, and flash-cooled in liquid nitrogen. X-ray diffraction datasets were collected at the beamlines BL45XU in SPring-8 and X06SA in Swiss Light Source. Diffraction data were processed with X-ray detector software (XDS) (27). The initial phases were obtained using the molecular replacement method with the AlphaFold2 predicted ApAgt structure AF-B3H2N1-F1 as the search model, using the MolRep program in the CCP4 suite (28). Further model refinements were performed using Refmac5 (29) and Coot (30). The refinement and manual rebuilding were reiterated until acceptable *R*_work_ and *R*_free_ values were obtained. The summary of the data collection and refinement statistics is given in Table 1.

### Preparation of acceptor glycopeptides

The peptide used in the glucosyl transfer assay was custom-synthesized (Toray Research Center). The amino acid sequence is Ac-Ala-Ala-Tyr-Asn-Val-Thr-Lys-Arg-(Lys- carboxytetramethylrhodamine [TAMRA])-COOH, in which the N-glycosylation sequon is underlined. The N-terminal α-amino group is modified with an acetyl group (Ac-). A 5(6)-TAMRA fluorophore is attached to the side chain ε-amino group of the C-terminal lysine residue for detection (31). The ApNGT protein was used to transfer a glucose residue to the asparagine residue in the sequon to prepare an acceptor glycopeptide, Ac-AAYN(Glc)VTKR(K-TAMRA), which is designated as the Glc_1_-peptide. In brief, the synthesized peptide was mixed with the ApNGT protein and UDP-Glc in TBS buffer (50 mM Tris–HCl, pH 7.4, 150 mM NaCl). After an overnight incubation at 37 °C, the reaction mixture was loaded onto a reversed-phase 5C18 AR-ii 10/250 column (Nacalai Tesque). Solvent A was 0.1% TFA in water and solvent B was 0.1% TFA in acetonitrile. A linear gradient of solvent B was applied from 20%B to 30%B. The glycopeptide fractions were pooled, dried with a SpeedVac concentrator, and resuspended in water. The purified glycopeptide solution was mixed with a saturated matrix αCHCA solution at a ratio of 1:1 (v/v). MALDI-TOF-MS spectra were acquired in the positive ion reflection mode using an AXIMA Performance spectrometer (SHIMADZU).

### Preparation of acceptor oligosaccharides

Five nanomoles of each oligosaccharide was dissolved in water and dried with a SpeedVac concentrator. The reducing ends of oligosaccharides were derivatized with 2-AP, as described (32). The reaction mixture was loaded onto a MonoFas silica gel spin column (GL Sciences) to remove the excess 2-AP. The PA-labeled oligosaccharides eluted from the spin column were diluted with water and stored at −20 °C until use. The amounts of the PA-labeled oligosaccharides were quantified by comparing the fluorescence intensity with that of Glc_6_-PA (2 pmol μl^−1^) in the PA-Glucose Oligomer (TAKARA 4108). The chemical structures of PA-labeled oligosaccharides were drawn using the ACD/Labs ChemSketch software (www.acdlabs.com) (Fig. S2).

### Assay of the glucosyl transfer activity of the AmGGT protein

In the assay with the Glc_1_-peptide as an acceptor, the enzyme concentration was 2.5 μM in a final volume of 10 μl. The Glc_1_-peptide and UDP-Glc were added to the reaction mixture at final concentrations of 1 μM and 1 mM, respectively. The glucosylation reaction proceeded at 37 °C for 1 h. The reaction mixture was loaded onto an AdvanceBio Glycan Mapping column (Agilent). Solvent A was 100 mM ammonium acetate buffer, pH 4.5, and solvent B was 100% acetonitrile. A linear gradient of solvent B was applied from 75% B to 59% B. In the assay with the PA (pyridylamino)-labeled oligosaccharides as acceptors, the enzyme concentration was 2.5 μM in a final volume of 10 μl. PA-labeled oligosaccharides and UDP-Glc were added at final concentrations of 4 μM and 1 mM, respectively. The glucosylation reaction proceeded at 37 °C for 1 h. The reaction mixture was loaded onto an AdvanceBio Glycan Mapping column (Agilent). Solvent A was 100 mM ammonium acetate buffer, pH 4.5, and solvent B was 100% acetonitrile. A linear gradient of solvent B was applied from 80%B to 54%B. For the assay with eukaryotic high-mannose type *N*-glycans as acceptors, PA-labeled M9 *N*-glycan was purchased from Masuda Chemical Industries (PA-033, Takamatsu), and PA-labeled G3M9 *N*-glycan was prepared as described previously (32). Briefly, *Saccharomyces cerevisiae* strain BY4741 was cultured, and the lipid-linked oligosaccharides were extracted from the yeast cells. The G3M9 oligosaccharide was generated by acid hydrolysis. After PA labeling, the spin column eluate was mixed with acetonitrile at a final concentration of 95% (v/v). The mixture was centrifuged at 15,000*g* for 10 min, and the supernatant was discarded. The pellet containing G3M9-PA was resuspended in 20 mM ammonium formate, pH 4.5, and 62% acetonitrile. The suspension was loaded onto a normal-phase Sugar-D 4.6/250 column (Nacalai Tesque). The mobile phase was 20 mM ammonium formate, pH 4.5, and 62% acetonitrile. The peak fractions were pooled, dried, and resuspended in water. The glucosyl transfer to the PA-labeled eukaryotic *N*-glycans was measured with 10 μM of the AmGGT protein in a final volume of 10 μl. The PA-labeled G3M9 or M9 oligosaccharides and UDP-Glc were mixed at final concentrations of 4 μM and 1 mM, respectively. The glucosylation reaction proceeded at 37 °C for 16 h.

The enzymatic activity was assessed in the presence of various UDP-sugars as donors. The reaction mixture contained the AmGGT protein, a UDP-sugar (donor), and Glcα1-6Glc-PA (acceptor) at final concentrations of 2.5 μM, 1 mM, and 4 μM, respectively, in a final volume of 10 μl. The glucosylation reaction proceeded at 37 °C for 16 h. The reaction products were separated by normal-phase chromatography, collected, and dried with a SpeedVac concentrator. The dried samples were dissolved in water and mixed with a saturated matrix 2,5-dihydroxybenzoic acid solution at a ratio of 1:1 (v/v). MALDI-TOF-MS spectra were acquired in the positive ion reflection mode using an AXIMA Performance spectrometer (SHIMADZU).

Dextranase (1,6-α-D-glucan 6-glucanohydrolase) purchased from Sigma-Aldrich (D5884) was used to estimate the linkage type of the glycosidic bonds in oligosaccharides produced by the AmGGT catalysis. The AmGGT protein was incubated with UDP-Glc as the donor and Glcα1-4Glc-PA as the acceptor at 37 °C for 1 h. The reaction was stopped by heat inactivation at 80 °C for 10 min. Dextranase was added to the reaction mixture at a final concentration of 5 units ml^−1^ and incubated at 37 °C for 16 h. The reaction products were separated by normal-phase chromatography as described above.

### UDP-Glo glycosyltransferase assay

The commercial UDP-Glo glycosyltransferase assay kit was purchased from Promega. The reaction mixture was dispensed into a 96-well plate (white, flat-bottom, nonbinding surface, Corning Catalog No. 3992). After the glycosyltransferase reaction, free UDP was converted into ATP, which was then converted into a luminescent signal by the luciferase reaction. The UDP detection reagent was prepared according to the manufacturer’s protocol. Luminescence proportional to the glycosyltransferase activity was measured using an Enspire plate reader (PerkinElmer). The reaction mixture contained the AmGGT protein, UDP-Glc (donor), and a substrate sugar (acceptor) at final concentrations of 25 nM, 100 μM, and 1 mM, respectively. The glucosylation reaction was initiated by adding the substrate sugar and proceeded at 37 °C for 1 h. Statistical analyses were performed with the EZR statistical software (www.jichi.ac.jp/saitama-sct/SaitamaHP.files/statmedEN.html) (33), an open-source statistical software program that is based on R version 4.3.1 (R Foundation for Statistical Computing) and R Commander (34). Data were analyzed by one-way ANOVA with the Dunnett *post hoc* test for multiple comparisons.

## Data availability

All data is included in the article and the Supporting information.

## Supporting information

This article contains Supporting information.

## Conflict of interest

The authors declare that they have no conflicts of interest with the contents of this article.
