# Supplementary material for: An uncharacterized gene from the Actinobacillus genus encodes a glucosyltransferase with successive transfer activity and unique substrate specificity
Source: J Biol Chem. 2025 Apr 30;301(6):108567. doi: 10.1016/j.jbc.2025.108567 (PMC12159676; doi:10.1016/j.jbc.2025.108567)
Supplement: Supporting information [file mmc1.docx]

Supporting Information

**An uncharacterized gene from the *Actinobacillus* genus encodes a glucosyltransferase with successive transfer activity and unique substrate specificity**

Takahiro Yamasaki^1,^*, and Daisuke Kohda^2^

**List of Material included:**

Figure S1. Phylogenetic analysis of 16S rRNA sequences from 18 representative strains belonging to the *Actinobacillus* genus.

Figure S2. Multiple sequence alignment of AmGGT and closely related glycosyltransferases.

Figure S3. Chemical structures of the 2-aminopyridine labeled disaccharides.

Figure S4. Effect of isomalto-oligosaccharide length on UDP-Glc hydrolysis by AmGGT.

Figure S5. CBB (Coomassie Brilliant Blue)-stained SDS-PAGE gel image.

Figure S6. Effects of metal ions and reaction solution pH on AmGGT activity.

Figure S7. The AmGGT protein was incubated with eukaryotic *N*-glycan, G3M9-PA, as the acceptor.

**Figure S1. Phylogenetic analysis of 16S rRNA sequences from 18 representative strains belonging to the *Actinobacillus* genus.** The dendrogram was constructed using the neighbor-joining method in the program MAFFT. The bootstrap values are displayed for each node as percentages > 50% for 100 trees.

**Figure S2. Multiple sequence alignment of AmGGT and closely related glycosyltransferases.** Multiple sequence alignment of the AmGGT and other five proteins that were top-ranked hits in the Blastp search using the AmGGT sequence as a query. The colored characters indicate the strictly conserved amino acid residues. The key amino acid residues focused on in this study are marked. The sequence of the AmGGT protein is numbered every 60 residues.

**Figure S3. Chemical structures of the 2-aminopyridine labeled disaccharides.** The structural formulas of the five disaccharides tested as acceptor substrates are shown.

**Figure S4. Effect of isomalto-oligosaccharide length on UDP-Glc hydrolysis by AmGGT.** Note that the acceptor molecules used did not contain a fluorescent PA group. The hydrolysis of UDP-Glc was measured using the UDP-Glo method, which converts UDP to ATP and measures luminescence produced by the luciferase reaction. The luminescence intensities of Glc_3_ (isomaltotriose), Glc_4_ (isomaltotetraose), and Glc_5_ (isomaltopentaose) were normalized to that measured with Glc_2_ (isomaltose) as the acceptor. Circles represent data points obtained from three independent experiments. The bar heights indicate the mean values. Statistical analyses were conducted by one-way ANOVA followed by Dunnett's two-sided post hoc test with Glc_2_ as the reference standard. **: p <0.001, n.s.: non-significant

**Figure S5. CBB (Coomassie Brilliant Blue)-stained SDS-PAGE gel image.** After separation by gel-filtration chromatography, the wild-type and mutant AmGGT proteins were separated on a 10%-20% gradient polyacrylamide gel under non-reducing conditions and then subjected to CBB staining. The molecular masses (kDa) of the protein size markers are shown.

**Figure S6. Effects of metal ions and reaction solution pH on AmGGT activity.** *A*, assays in the presence of various metal ions at a concentration of 10 mM each. *B*, buffer adjusted to the indicated pH was added to the reaction mixture at a final concentration of 50 mM. In *A* and *B*, the reaction mixtures contained UDP-Glc as the donor and Glcα1-6Glc-PA as the acceptor. The reaction products were separated by normal-phase UPLC chromatography, and the fluorescence intensity of the pyridylamino group was measured. Asterisks indicate positions of non-specific peaks. Arrows indicate reaction products.

**Figure S7. The AmGGT protein was incubated with eukaryotic *N*-glycan, G3M9-PA, as the acceptor.** *A*, normal-phase chromatograms of the reaction mixtures containing G3M9-PA after 16-h incubation with/without the AmGGT protein. The donor was UDP-Glc. The G3M9 glycan structure is shown. The blue squares represent GlcNAc, the green circles represent Man, and the blue circles represent Glc. *B*, MALDI-TOF MS spectra in the positive ion mode, using DHB as the matrix. The *m*/*z* values of the observed peaks are indicated. The double-headed arrow indicates the difference in the *m/z* value from the G3M9-PA peak. The observed peak*, m*/*z* 2649.24, was assigned to Glc-G3M9-PA (a Glc adduct of G3M9-PA, calculated *m*/*z* 2648.88 [M+H+K]^+^).
